# Assessment of insulin-degrading enzyme inhibitor for the treatment of corneal erosion in a rat model

**DOI:** 10.1007/s00417-024-06717-1

**Published:** 2024-12-23

**Authors:** Levy Issac, Dollberg Dolev, Bahar Irit, Dotan Assaf

**Affiliations:** 1 Department of Ophthalmology and Laboratory of Eye Research, Rabin Medical Center – Beilinson Hospital, Felsenstein Medical Research Center , 39 Jabotinski St., Petach Tikva, 49100 Israel; 2 Faculty of Medicine, Tel Aviv University, Tel Aviv, Israel

**Keywords:** Chronic corneal erosion, Corneal epithelial defect, Keratopathy, Corneal wound healing, Insulin-degrading enzyme, IDE

## Abstract

**Background:**

Diabetes poses a risk to diabetic keratopathy in up to two-thirds of patients. Insulin-degrading enzyme (IDE) is a protease that can break down insulin and several growth factors and may impair wound healing. Increased IDE levels have been found in fluid from diabetic skin ulcers. This study sought to determine the effect of IDE inhibitor on corneal wound healing in a rat model.

**Methods:**

Thirty-four male Wistar rats were divided into two groups: no diabetes and streptozocin-induced diabetes. Six weeks later, a 4-mm central corneal erosion was created under anesthesia in the right eye of all rats. In each group, half the rats were treated with ADT21 drops (IDE inhibitor) and half with NaCl 0.9% (sham) drops, four times daily. Image J analysis was performed to evaluate the area of erosion and healing rate.

**Results:**

There was a trend for more rapid healing in rats treated with IDEI than NaCl drops, regardless of the diabetic condition. Comparison of erosion closure over time revealed that the wounds closed significantly more quickly in the non-diabetic rats treated with IDEI than in the non-diabetic rats treated with NaCl (*p* = 0.045), overall mean closure time 56.00 h, 95% CI [50.54, 61.46]. No such difference was seen in the diabetic group.

**Conclusions:**

To our knowledge, this is the first study to test ADT21 drops as a novel treatment for corneal wound repair. Our results suggest a potential benefit of IDE inhibitor for treating corneal injury.

## Abstract (highlights)

**Key messages:**

*****What is known?***:**

- Chronic corneal erosions can be difficult to manage, and over the years, various drugs and substances have been tried as a solution to cure chronic corneal erosions.
- Several studies in humans reported a beneficial effect of topical insulin drops in cases of persistent epithelial defects of various etiologies.

*****What is new?***:**

- We sought to investigate the effect of novel eyedrops containing Insulin- degrading enzyme inhibitor, which can potentially increase insulin and various growth factors in the eye and enhance corneal wound healing.
- Our results suggest a potential benefit of insulin-degrading enzyme inhibitor for treating corneal injury.

## Introduction

Diabetes mellitus is a pandemic. According to the World Diabetes Organization, there were over 400 million people with diabetes in 2015, and that number is expected to exceed 640 million in 2024 [1]. Microvascular damage secondary to hyperglycemia, particularly in the retina, is the most common complication. Diabetes can also affect anterior segment structures in the eye, including the cornea, conjunctiva, and lacrimal gland [1]. In the cornea, the microvascular changes result in a decrease in the supply of oxygen and nutrients to the nerve fibers and, consequently, a decrease in nerve fiber concentration and corneal sensation. Injury to the sub-basal nerve plexus between the epithelial layer and Bowman membrane may be observed by confocal microscopy [2]. According to the literature, keratopathy may develop in up to two-thirds of patients with diabetes. The damage to the basal epithelial cells and decrease in corneal innervation can lead to the formation of epithelial defects and impaired wound healing, increasing the risk of corneal infection [3, 4]. It has been shown that diabetes is associated with recurrent corneal erosion, chronic corneal erosion, and dry eye [5–7].

In 1949, the discovery of insulin-degrading enzyme (IDE), a protease that breaks down the B chain of the insulin hormone [8], prompted studies of the potential use of IDE inhibitors (IDEIs) to control insulin levels in animal models of diabetes. One of the earlier reports found that endogenous IDEI isolated from the liver successfully lowered and balanced glucose levels in rats and rabbits [9]. The results were supported by more recent findings of increased plasma insulin levels and improved glucose clearance in obese and lean mice treated with IDEI for a short period [10]. It was recently published that a novel IDE inhibitor has shown efficacy both in type one and type two diabetic mouse models when given over the course of 15 to 30 weeks. The treatment shows efficacy both in males and females given nasally [11].

IDE was reported to be elevated in pre-diabetic patients [12]. Others showed that IDE enzyme activity is increased in wound fluid derived from peripheral diabetic skin ulcers [13]. A high concentration of IDE may induce the breakdown not only of insulin but also of various growth factors, thereby impairing wound healing. Insulin is known to act in synergy with platelet-derived growth factor and epidermal growth factor (EGF) to increase tissue granulation and collagen deposition [14, 15]. EGF, among other growth factors, has been shown to stimulate corneal epithelial cell migration both in vivo and in vitro [16]. The EGF receptor is also found in the cornea and is thought to be a key component in the process of corneal epithelial layer healing and reformation of cell-to-cell junction [2]. High glucose levels can impair EGF receptor signaling [17].

Immunochemistry studies of the human eye identified insulin in the tear film and alpha and beta subunits of insulin receptors in various eye parts, including the cornea [16, 18]. In diabetic rodents, treatment of corneal erosion with both systemic and topical insulin showed an advantage of glucose balance and topical insulin treatment in promoting epithelial regeneration [19, 20]. Moreover, several studies in humans reported a beneficial effect of topical insulin drops in cases of persistent epithelial defects of various etiologies [21–24].

Over the years, various drugs and substances have been tried as a solution to cure chronic corneal erosions; among the substances that showed effectiveness were neuropeptides, anti-inflammatory drugs, autologous serum, and opioid antagonists [20, 25, 26]. Based on the assumption that increased activity of IDE enzyme in the eyes of diabetic rats may inhibit the healing of corneal erosions, we sought to investigate the effect of eyedrops containing IDEI on the duration and rate of corneal wound healing in a diabetic rat model.

## Materials and methods

### Experimental animals

Thirty-six male Wistar rats weighing 275–295 g were obtained from Envigo RMS Laboratories, Israel. The rats were maintained and handled according to the recommendations of the Association for Research in Vision and Ophthalmology Statement for Use of Animals in Ophthalmic and Visual Research. The experimental protocol was approved by our institutional Animal Care and Use Committee of Rabin Medical Center (approval #02720).

### Induction of diabetes

All rats were weighed, and basal blood glucose level was quantified with a glucometer (Performa, Accu-Chek) from venous blood drawn from the tail. Nineteen rats were selected at random to undergo induction of diabetes type 1 by intravenous injection of 60 mg/kg of streptozocin (Merck KGaA, Darmstadt, Germany) in ice-cold citrate buffer pH 4.4 [27]. The procedure was performed under inhalational anesthesia with isoflurane using a glass dome.

Before the corneal wound was induced, blood glucose levels were monitored every two days for the first week and once again after six weeks. A glucose level of over 200 mg/dL was considered diabetes mellitus (Gheibi et al. 2017) [28].

The remaining (non-diabetic) rats served as the control group.

### Corneal erosion

Two rats died during the induction of diabetes, leaving 17 rats in each group.

Six weeks after induction of diabetes, all rats were placed under anesthesia with isoflurane gas and topical oxybuprocaine hydrochloride 0.4% drops (Localin, Fischer Pharmaceuticals, Bnei Brak, Israel) applied in the right eye. With the aid of a surgical microscope, a corneal wound was created by placing an incision in the epithelium using a disposable biopsy punch 4 mm in diameter (Uni-Punch^®^, Premier, Plymouth Meeting, PA, USA) and then softly pilling the epithelium with a no.11 surgical scalpel blade (Swann-Morton, Sheffield, England).

### Care and observation

IDEI drops were concocted with ADT21 peptide (GenScript, Inc. and the Blavatnik Centre for Drug Discovery, Tel Aviv University, Tel Aviv, Israel) [11]. The peptide was dissolved in dimethyl sulfoxide (DMSO) 0.02% and diluted in sodium chloride (NaCl) 0.9% to a concentration of 2 mmol/L and kept at −20 °C. Control drops were prepared from NaCl 0.9% mixed with DMSO 0.02%. [The DMSO was mixed with NaCl in light of reports that at high concentrations, DMSO may benefit corneal epithelial healing [29].] In each group of 17 rats, nine were treated with IDEI drops, and eight were treated with NaCl (sham) drops. Drops were administered four times daily, at 09:00, 13:00, 17:00, and 21:00. Eyes were examined with a slit-lamp twice daily until complete healing. The epithelial defect was stained with topical fluorescein (Fluoro Touch, Fluorescein Sodium Ophthalmic Strips, Madhu Instruments, New Delhi. India) and photographed under cobalt blue light. The eyes were examined and photographed with a slit lamp twice a day until complete healing. The area of corneal erosion was calculated and analyzed using ImageJ (Rasband, W.S., ImageJ, U. S. National Institutes of Health, Bethesda, MD, USA; https://imagej.nih.gov/ij/, 1997–2018).

### Statistical analysis

Groups were compared for time to complete healing using a t-test for independent samples and for a percentage of rats that achieved complete wound closure at the different time points by Fisher’s exact test. The log-rank (Mantel-Cox) test was used to compare rates of complete closure over time. The data was tested for normal distribution. Statistical significance was defined as *p* < 0.05.

## Results

Of the total 34 rats included in the study, 17 underwent induction of diabetes mellitus, and 17 were not diabetic. Nine rats in each group were treated with the IDEI regimen, and eight were treated with NaCl (sham) eye drops. A non-healing ulcer formed in 7 (41.2%) of the diabetic group compared to 2 (11.8%) of the non-diabetic group (*p* = 0.118), regardless of the type of treatment. Eyes with a sustained corneal infiltrate (*n* = 9) were removed from the analysis.

## Time to reepithelization

Within the non-diabetic group, there was a trend towards faster healing in the IDEI–treated subgroup (mean 51 ± 8.49 h) than the NaCl-treated subgroup (61.71 ± 10.797 h), but the difference did not reach statistical significance (*p* = 0.051), mean difference 10.71, 95% CI [−0.43, 21.472]. In the diabetic rats, however, the result was unexpected: the recovery time was not shorter with IDEI drops. Healing times in the treatment subgroups of the diabetic rats were 64 ± 18.07 h and 57 ± 11.49 h, respectively (*p* = 0.52), mean difference − 7.00, 95% CI [−30.70, 16.7].

### Rate of complete healing

**Fig. 1:**
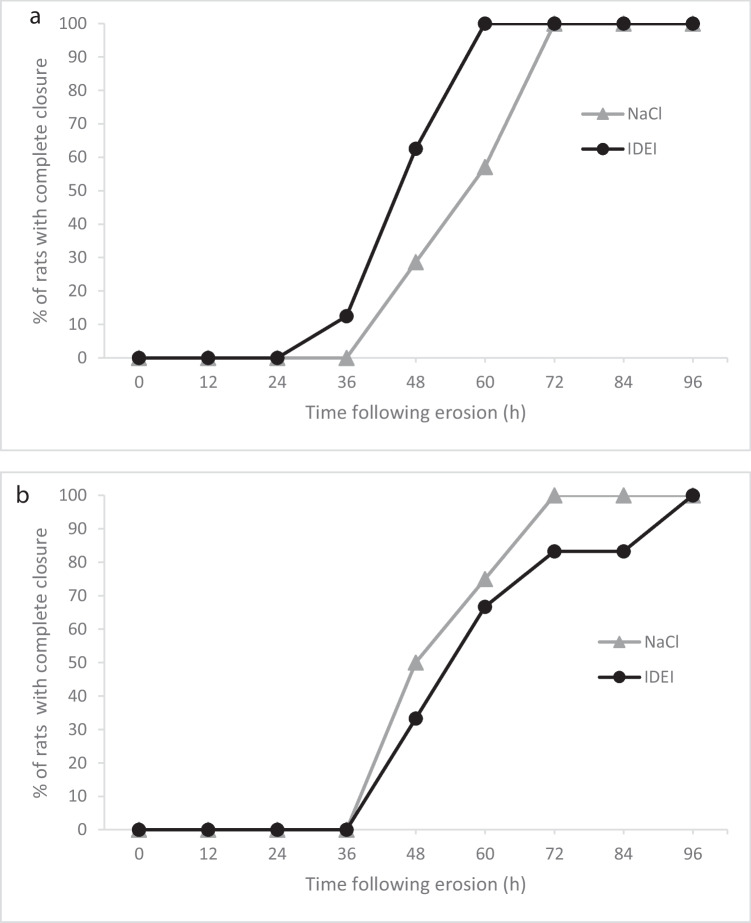
Percentage of non-diabetic rats (a, n=15), and diabetic rats (b, n=10) with completely healed erosions per time point. Measured with Fisher’s exact test

In the group of diabetic rats, there was no significant difference at any time point between the treatment subgroups in the percentage of eyes with completely healed erosions. In the non-diabetic group, however, at the 60-hour time point, a higher percentage of healed erosions was observed in the IDEI-treated than in the NaCl-treated subgroup (100% vs. 57.1%, *p* = 0.077). Figure 1a and b show the percentage of rats with completely healed erosions at each time point in the non-diabetic and diabetic groups, respectively. The 60-hour time point is not clinically significant in treating corneal erosion, but in our analysis, it was the time point where a major difference was found between groups.

Overall, there was a trend for more rapid healing in rats treated with IDEI than NaCl drops, regardless of the diabetic condition. After 60 h, the erosion fully closed in 85.7% of the IDEI-treated rats and 63.6% of the NaCl-treated rats (*p* = 0. 350).

After excluding the rats with infected corneal ulcers, seven diabetic rats, and two non-diabetic rats, all erosions in the study rats entirely healed by 96 h.

**Fig. 2:**
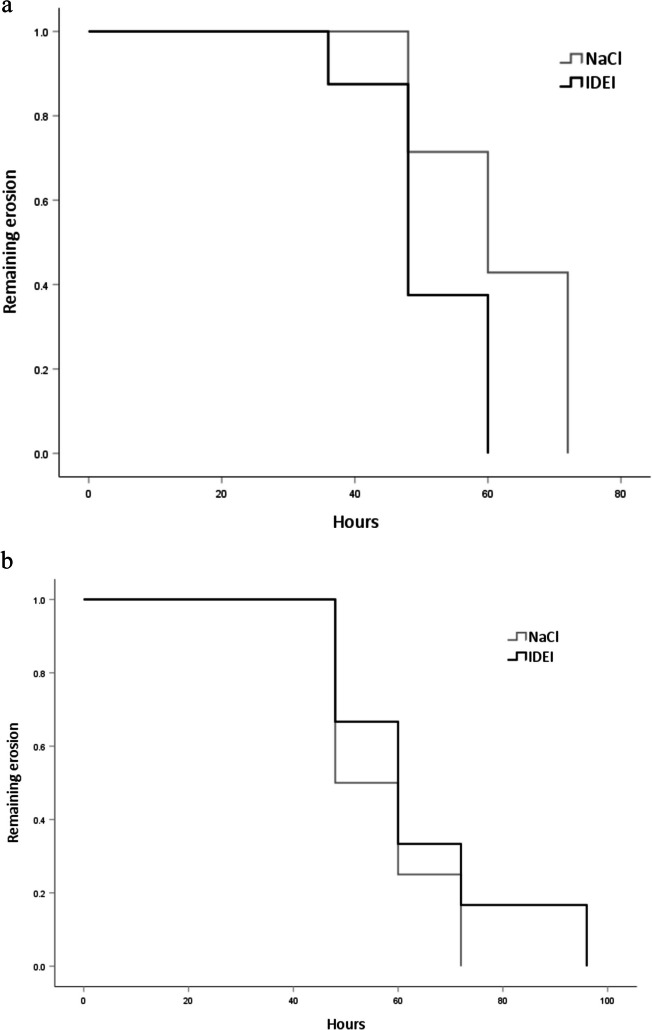
Percentage of non-diabetic rats (a, n=15) and diabetic rats (b, n=10) in which corneal erosions remained over the treatment course. Survival analysis using the log rank test was used. The rate of closure was faster in the IDEI group within non-diabetic rats (p=0.045), but no difference was observed in the diabetic rats

**Fig. 3:**
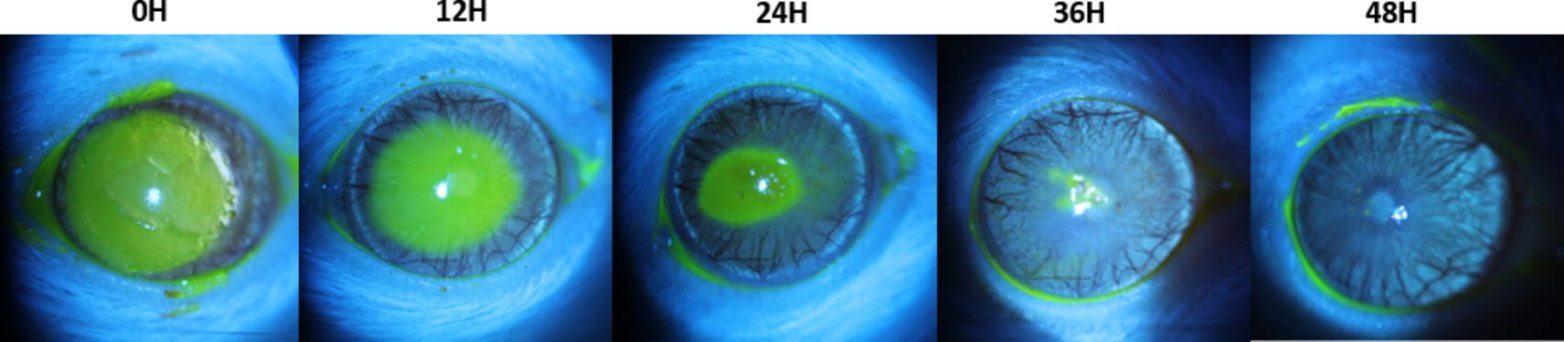
Slit-lamp photos of the cornea of a diabetic rat at different time points, from total erosion to complete healing under IDEI drops

**Fig. 4:**
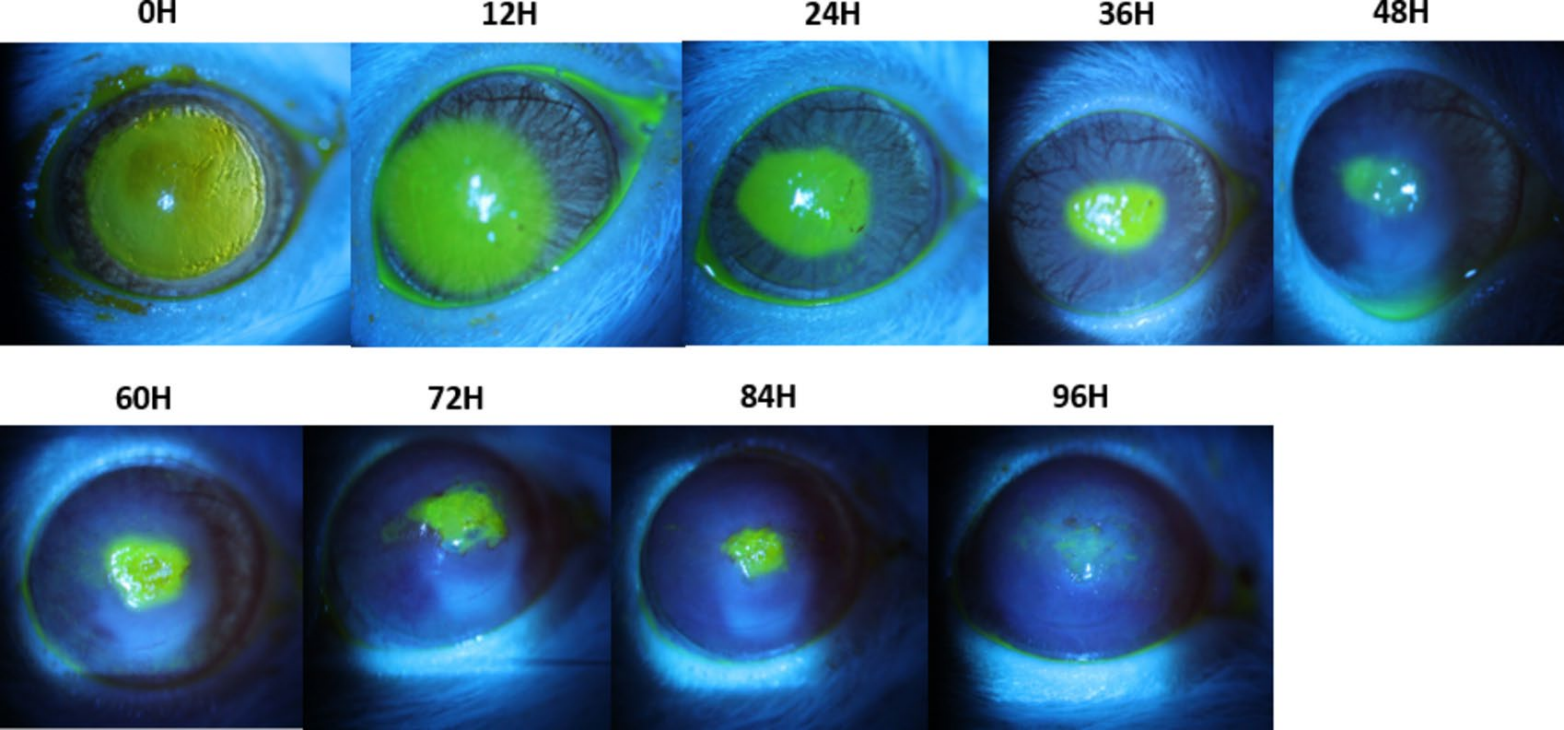
Slit-lamp photos of the cornea of a diabetic rat under treatment of NaCl drops, with a non-healing ulcer

Comparison of erosion closure over time revealed that the wounds closed significantly more quickly in the non-diabetic rats treated with IDEI than in the non-diabetic rats treated with NaCl (Fig. 2a, *p* = 0.045), overall mean closure time 56.00 h, 95% CI [50.54, 61.46]. No such difference was seen in the diabetic group (Fig. 2b). Representative slit-lamp photos taken under cobalt blue light are shown in Figs. 3 and 4.

## Discussion

Diabetes may be complicated by chronic wounds and poor wound healing. The risk of chronic foot ulcer during the lifetime of a patient with diabetes can reach up to 25% [13, 30, 31]. Studies have attempted to improve and accelerate wound healing by increasing insulin and growth factor levels in wound fluid using several treatments, including IDE inhibitors [31, 32]. In the eye, high glucose levels can impair corneal epithelial healing and lead to recurrent and chronic corneal erosions [3].

This study aimed to seek a novel drug for the treatment of corneal erosions and chronic corneal wounds due to high glucose levels. We hypothesized that IDE, a known protease that can break down insulin and several growth hormones that participate in wound healing, may serve as a good candidate, given the effect of these factors on corneal re-epithelization. To the best of our knowledge, this is the first study to test IDEI drops for corneal wound repair. Studies have shown that besides brain tissue, IDE is expressed in the retina [33], but our search of the literature yielded no studies of its presence or effect in the anterior chamber of the eye.

We used streptozocin for diabetes induction. Akbarzadeh et al. (2007) reported that streptozocin caused the destruction of the beta cells in the islets of Langerhans, leading to the development of clinical diabetes within 2–4 days of administration. In a study of diabetic rats, Yin et.

al. (2011) demonstrated lens opacity after three weeks of hyperglycemia and cataracts in nearly all cases by eight weeks. There was also a minor yet significant decrease in corneal sensitivity, and thinner and fewer corneal nerve fibers could be seen by confocal microscopy. Accordingly, prior studies reported a significant delay in epithelial wound healing 4 to 8 weeks after the onset of diabetes mellitus [2, 20]. Based on these data, we set an interval of 6 weeks between diabetes induction and the creation of a 4-mm central corneal erosion in the right eye of all rats.

The limitations of this study are the small study group which restricted the statistical analysis. The fact that the rats were not treated with a topical antibiotic may have led to a large number of infected corneal ulcers that did not achieve complete healing and, consequently, a smaller group of diabetic rats that were eligible for final analysis. Because of the difficulty in creating and maintaining an infection-free environment, preventive topical antibiotic treatment should be considered in future studies, as reported by others [19, 20, 29, 34, 35].

In conclusion, the results of the present study suggest that IDEI drops may support corneal healing. Further research is needed on a larger number of eyes to determine if different IDEI concentrations and application frequencies would yield a greater beneficial effect. We plan to investigate the presence of IDE enzymes and receptors in the anterior segment of the eye. Overall, this study highlights the importance of continued research on the ophthalmic effects of diabetes and the potential for novel treatments.

## Declarations

This Research involving Animals. The experimental protocol was approved by our institutional Animal Care and Use Committee of Rabin Medical Center (approval #02720). Animals were housed in appropriate conditions, and procedures were designed to minimize pain, distress, and suffering.
