# Correction: Assessment of insulin-degrading enzyme inhibitor for the treatment of corneal erosion in a rat model

**DOI:** 10.1007/s00417-025-06932-4

**Published:** 2025-07-25

**Authors:** Levy Issac, Dollberg Dolev, Bahar Irit, Dotan Assaf

**Affiliations:** 1 Department of Ophthalmology and Laboratory of Eye Research, Rabin Medical Center– Beilinson Hospital, Felsenstein Medical Research Center, 39 Jabotinski St., Petach Tikva, 49100 Israel; 2 Faculty of Medicine, Tel Aviv University, Tel Aviv, Israel

**Correction: Graefe's Archive for Clinical and Experimental Ophthalmology (2024) 263:1015-1021**

10.1007/s00417-024-06717-1

In the published original article, the author name “Levy Issac“ appeared twice in the author byline.

It should be corrected from:

Levy Issac^1,2^, Dollberg Dolev^1,2^, Bahar Irit^1,2^, Dotan Assaf^1,2^, Levy Issac^1,2^

To:

Levy Issac^1,2^, Dollberg Dolev^1,2^, Bahar Irit^1,2^, Dotan Assaf^1,2^

The original article has been corrected.

